# Echocardiographic Assessment of Left Ventricular Function 10 Years after the Ultra-Endurance Running Event Eco-Trail de Paris^®^ 2011

**DOI:** 10.3390/ijerph19148268

**Published:** 2022-07-06

**Authors:** Romain Jouffroy, Oussama Benaceur, Jean-François Toussaint, Juliana Antero

**Affiliations:** 1 Intensive Care Unit, Ambroise Paré Hospital, Assistance Publique—Hôpitaux de Paris, 92100 Boulogne-Billancourt, France; oussama.benaceur@aphp.fr; 2 IRMES—Institute for Research in Medicine and Epidemiology of Sport, INSEP—National Institute of Sport, Expertise, and Performance, 75012 Paris, France; jean-francois.toussaint@aphp.fr (J.-F.T.); juliana.antero@insep.fr (J.A.); 3 INSERM U-1018, Centre de Recherche en Epidémiologie et Santé des Populations, Paris Saclay University, 94800 Paris, France; 4 URP 7329, Université de Paris, 75012 Paris, France; 5 Centre d’Investigations en Médecine du Sport, Hôpital Hôtel-Dieu AP-HP, 75004 Paris, France

**Keywords:** long-distance running, long term, echocardiography, alteration

## Abstract

*Background:* Regular and moderate physical activity is beneficial for physical and mental health, resulting in an increase in life expectancy for both sexes. From a cardiovascular point of view, although the benefits of regular moderate physical exercise have been established, the long-term effects of repeated ultra-endurance running events are still unknown. *Hypothesis:* The aim of our study is to evaluate the 10-year evolution of the parameters of the left ventricular systolic and diastolic functions of amateur subjects regularly practising ultra-endurance running events using resting echocardiography. *Study design:* Cross-sectional study. *Level of evidence:* Level 3—non-randomized controlled cohort/follow-up study. *Methods:* The 66 participants who participated in the 2011 edition of the Eco-Trail de Paris^®^ were contacted by e-mail. Demographic data, sports practice, and the results of an echocardiography scan carried out during the year 2021 evaluating left ventricular systolic and diastolic function variables were collected. Echographic variables from 2011 and 2021 were compared using the paired Student’s *t*-test. *Results:* Forty-six (70%) participants responded positively. Twenty (30%) participants could not be reached and were not analysed. Of the 46 respondents, 42 (91%) provided data from a trans-thoracic cardiac ultrasound performed in 2021. Over the past 10 years, the participants reported having completed an average of 4 ± 2 ultra-trails per year. No significant differences were observed between left ventricular diastolic and systolic echocardiographic parameters between the years 2011 and 2021. *Conclusions:* Among amateur participants, long-distance running is not associated with an alteration in the echocardiographic parameters of resting left ventricular systolic and diastolic function after 10 years of practice. *Clinical relevance:* Long-term long-distance running practice is not associated with left ventricular cardiac function alteration. These results suggest a potential adaptation role of the cardiovascular system to regular and moderate long-distance running practice.

## 1. Introduction

Physical activity is a major determinant of health status and physical condition and of cardio-respiratory capacity, muscular condition, and the maintenance of autonomy with advancing age in particular, but also of the quality of life at any age. In the general population, regular and moderate physical activity is beneficial for health and the extension of life expectancy for both sexes [1], with a dose–response relationship between the amount of physical activity and the associated reduction in all-cause mortality [2,3]. This same relationship has been reported among high-level athletes [4,5,6,7,8,9,10,11,12,13]. In this regard, physical activity is involved in the primary, secondary, and tertiary prevention of many chronic diseases—in particular, cardiovascular diseases such as hypertension and coronary heart disease. Thus, regular physical activity is recommended to improve the length and quality of life [1,14,15,16,17,18,19,20].

From the cardiovascular point of view, while the benefits of regular moderate exercise are established [21], the effects of prolonged exercise are unknown because of the biochemical, electrocardiographic, and echocardiographic disturbances observed during prolonged exercise such as marathon running [22,23]. These disturbances include geometric changes [22,24,25] and disturbances of segmental kinetics [26].

After an ultra-endurance running event, i.e., a race lasting more than 6 h, we demonstrated the occurrence of transient left ventricular diastolic and systolic dysregulations with complete recovery 9 days after the end of the event [27]. We have also shown in previous studies that Olympian runners had a greater reduction in the risk of death from cardiovascular disease compared to Olympians practising other types of exercise [12]. However, no study has evaluated the clinical long-term cardiac echocardiographic impact of repeated ultra-endurance running events despite recent controversies [28,29,30]. Regardless, ultra-endurance running’s popularity has increased over the last two decades [29]; to date, no study has followed participants since their first involvement in the sport. Most studies investigating long-term health problems lack the crucial definition of dose and a clear definition of “chronic or repeated” participation [29,30].

The objective of our study is to evaluate the 10-year evolution of the parameters of the left ventricular systolic and diastolic functions of amateur subjects regularly practising ultra-endurance running events using resting echocardiography.

## 2. Methods

The 66 amateur participants, all male, who took part in the study assessing cardiac function by repeated echocardiographic examinations during the 2011 edition of the Eco-Trail de Paris^®^ [27] constituted the starting population for our study. Each runner received an e-mail specifying the nature and objective of the study. The 66 participants fulfilled the study’s inclusion criteria because of their participation in the 2011 study [27]. No runner with a medical history and in whom significant heart disease or abnormality in the echocardiographic examination was found prior to inclusion was included in the 2011 study [27].

All respondents completed a questionnaire collecting demographic data (age, sex, weight, height) and data on their sports practice over the past 10 years (number and hourly amount of weekly training sessions in km, length of time of practice for ultra-distance races, names and number of ultra-endurance races completed annually over the past 10 years). The questionnaire recorded health problems, particularly cardiovascular and surgical ones, that had occurred in the last 10 years as well as treatments taken in the last 10 years.

Participants who did not respond to the email for any reason (e.g., change of email address) were excluded because of the impossibility of reaching them. After being clearly informed of the study protocol, the participants freely consented to participate and to transmit their demographic data, information about their sports practice, and the results of an echocardiography scan performed during the year 2021 by the cardiologist of their choice.

The assessment of cardiac function was based on the left ventricular systolic and diastolic function variables, i.e., the numeric values provided by individual cardiologists. The variables of interest were (i) left ventricular end-diastolic parameters in two-dimensional mode: interventricular septal thickness, left ventricular posterior wall thickness, left atrial and left ventricular end-diastolic diameters, and aortic diameter, (ii) left ventricular end-systolic parameters in two-dimensional mode: left ventricular end-systolic area with a calculation of left ventricular ejection fraction, (iii) left ventricular Doppler measurements: subaortic velocity–time integral; aortic ejection flow and cardiac output; mitral and septal E, A, and e’ waves and mitral and septal a’ waves and S waves, and (iv) left ventricular parietal strain measurements: global and longitudinal strain (4, 3, and 2 cavity slices).

Categorical variables were expressed as absolute values with a percentage, continuous quantitative variables with Gaussian distribution by the means of standard deviation, and continuous quantitative variables with non-normal distribution by the median with interquartile range [1st quartile–3rd quartile]. Multiple imputations based on the Markov chain Monte Carlo method were performed for missing data from cardiac ultrasound scans performed in 2021 using the mice package method.

Comparisons between the variables of interest from the 2011 and 2021 echocardiography scans were performed using the paired Student’s t-test to account for the repeated-measure design of the study. All tests were two-tailed with a statistically significant *p*-value < 0.05. All analyses were performed using R 3.4.2 software (http://www.R-project.org; The R Foundation for Statistical Computing, Vienna, Austria).

The study protocol was approved by an independent ethics committee (Ethical Committee of the French Society of Anaesthesia and Intensive Care on 11 April 2021—IRB number 00010254-2021-049).

## 3. Results

**Figure 1:**
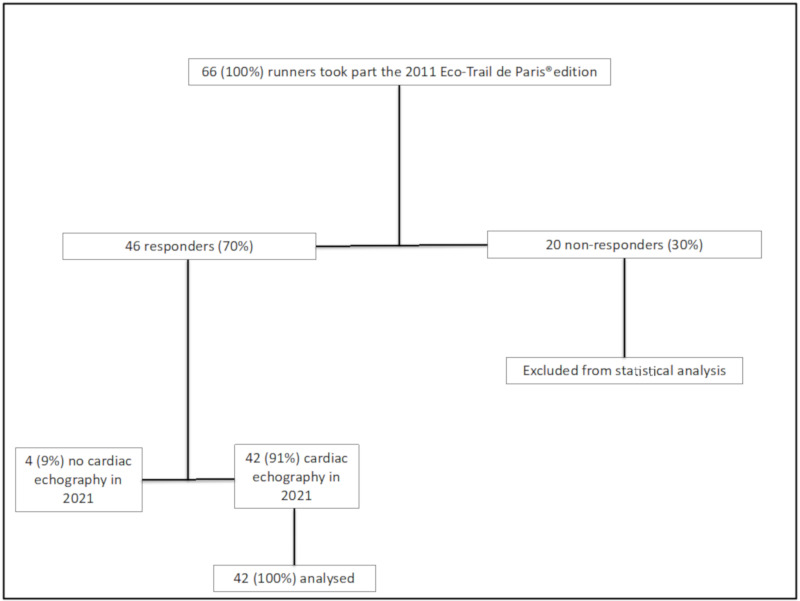
Study flow chart of the 66 participants involved in the 2011 Eco-trail de Paris^®^ edition that were contacted by e-mail.

Of the 66 participants who took part in the 2011 edition of the Eco-Trail de Paris^®^, 46 (70%) responded favourably and were included in the analysis. Twenty participants (30%) could not be reached and were not included in the analysis. Of the 46 respondents, 42 (91%) provided data from a trans-thoracic cardiac ultrasound performed during the year 2021. For the remaining four (9%) who did not perform an echocardiogram in 2021, the data were imputed (Figure 1).

The demographic characteristics of participants in 2011 and 2021 are summarised in Table 1.

Over the past 10 years, participants reported having completed an average of 4 ± 2 ultra-trails per year (min = 1 and max = 10). Annex reports indicate the competitions in which the participants participated in between 2011 and 2021.

The health problems reported were one case of arterial hypertension without the need for treatment (2%), one case of periostitis (2%), one case of tendonitis (2%), two cases of low back pain (4%), and one case of a meniscal tear that required surgery (2%). No runner reported cardiac rhythm disturbance.

The treatments reported were non-steroidal anti-inflammatory drugs for four participants (9%).

Four participants (9%) declared that their health problems had impacted their sports activity, and three (7%) declared that these problems were related to ultra-trail running. One runner (2%) stopped running ultra-trails in 2012 but continues to run as a leisure activity and was not included in the analysis.

No significant differences were observed between the echocardiographic parameters assessing end-diastolic and end-systolic geometric measurements or left ventricular diastolic and systolic function between the years 2011 and 2021 (Table 2).

## 4. Discussion

In this study, we reported the absence of alterations in the parameters of left ventricular systolic and diastolic cardiac functions evaluated by resting transthoracic echocardiography in amateur long-distance participants after 10 years of practice.

Although the beneficial effects of regular and moderate physical activity on health [21] are now well-known following studies carried out in the cardiovascular field in particular [28], the impact of long-term physical activity remains controversial [31]. The geometric changes in the heart observed by echocardiography immediately after a marathon [22,24,25] were not retrieved from the at-rest echocardiography results of amateurs who have been practising ultra-endurance running for the 10 years of our study. This suggests a determining role for the cardiovascular adaptation of the organism to the intensity of the exercise, including training, contrary to a previous narrative review report [26], despite its methodological flaws [30]. On the other hand, previous studies reported a negative relationship between cardiovascular health status and a sedentary lifestyle [29,31,32,33,34], which has been emphasized more recently after the COVID-19 lockdown [35].

In addition, during an ultra-endurance running event, transient left ventricular dysregulation, first diastolic and then systolic, was observed with resolution ad integrum on the 9th day post-exercise [27], suggesting that these transient modifications are more a result of transient fatigue than a definitive alteration in cardiac function. Although studies had suggested the possible occurrence of exercise-related myocardial infarction by measuring CPK [36,37], they were subsequently refuted by the results of muscle biopsies reporting the exclusively muscular origin of the observed increase in CPK levels [23,38,39].

Regular moderate physical activity has been reported to prevent and treat age-related degeneration [40,41]. From a physiological point of view, exercise reduces sarcopenia by decreasing inflammation [42]. In addition, it increases anabolism [43], protein synthesis [44], mitochondrial remodelling [45,46], and the appearance of fibro–adipogenic progenitors in response to exercise-induced muscle damage, allowing regenerative inflammation to improve muscle endurance [47].

Our results are consistent with the data in the literature reporting the absence of deleterious cardiovascular effects of moderate-intensity endurance exercise [48]. Contrary to what has been reported in studies concerning higher-intensity events [49,50,51,52], we did not observe any modifications in the cardiac morphological characteristics. In addition, we found the absence of alterations in the left ventricular systolic and diastolic functional parameters reported by other authors in the field of physical endurance exercise [49,53,54,55,56,57,58]. Our results corroborate the data showing a lack of a deleterious effect on cardiac remodelling and the occurrence of early cardiac failure; rather, it is a transient failure (24–48 h) with complete recovery, “cardiac fatigue” [27,51,59]. Although the beneficial dose–response relationship reported for moderate physical exercise is debated for prolonged intense physical exercise [48,60,61], our data are in favour of an adaptation mechanism to the latter, which may partly explain the increased longevity observed in the general population, high-level sportsmen, and women regularly practising endurance physical activity at moderate levels of intensity [12,13,28,62,63,64]. The absence of deterioration in the echocardiographic parameters observed in our study population is partly explained by the normality of the initial cardiac function of the subjects included.

However, this study has limitations. Our sample size is modest, and for 30% of the participants, we could not obtain information about their health status and were not able to clearly specify whether the absence of these respondents was random or not and therefore if it could have affected our results. The absence of deleterious echocardiographic effects does not mean that the regular ultra-trail practice is beneficial since we reported the absence of deterioration and not improvement in the echocardiographic parameters of left ventricular cardiac function—even if we could suppose an expected low level of deterioration since the participants had aged 10 years. In this study, we did not use the same echocardiograph that was used 10 years earlier. Moreover, we did not explore the echocardiographic parameters of right ventricular cardiac function. Our statistical approach is descriptive and not causal, so our results do not allow us to conclude that ultra-trail running is part of the inverse relationship described between the intensity of physical activity and the incidence of atheromatous cardiovascular disease [65,66,67,68]. On the other hand, our data correspond with the absence of an increase in risk in relation to sustained intense physical exercise, contrary to what has been reported by indirect indices not considering confounding factors [69]. Moreover, our study population is made up of subjects without previous cardiovascular pathologies, thus limiting the evaluation of the effect of regular ultra-trail practice on the health of patients with cardiovascular diseases, as reported in coronary and hypertensive patients [70]. Moreover, the echocardiographic examinations were carried out at rest, preventing any extrapolation of the evolution of the per-effort parameters. We evaluated the number, not the intensity, of ultra-trail races and training. Finally, our methodology does not allow for the consideration of other factors influencing cardiac function [71], especially data regarding heart valve function. Despite the fact that no other changes occurred, we cannot exclude the possibility that if participants had “upped their game” and substantially boosted the volume/intensity of their races and training, cardiac remodelling may have occurred [53,72].

However, to the best of our knowledge, this is the first study assessing the long-term cardiac effects among regular amateur long-distance running practice participants.

## 5. Conclusions

In amateur long-distance running participants, no alterations in the parameters of the left ventricular systolic and diastolic cardiac functions evaluated by resting transthoracic echocardiography were observed after 10 years of practice. Our results suggest a potential adaptation role of the cardiovascular system to regular and moderate long-distance running practice. Further studies are needed to confirm this preliminary data during exercise but also to specify the possible beneficial effect of regular long-distance running.

## Figures and Tables

**Table 1:** Characteristics of the 46 amateur participants. Data are expressed as mean ± standard deviation (SD) and range (min–max value).

	Year 2011		Year 2021	
Variable	Mean ± SD	Range (Min–Max Value)	Mean ± SD	Range (Min–Max Value)
Age (years)	43 ± 7	25–61	53 ± 7	42–71
Height (cm)	176 ± 7	167–188	177 ± 6	165–188
Weight (kg)	74 ± 8	61–82	69 ± 6	60–80
Body mass index (kg/m^2^)	22.4 ± 1.1	18.9–25.2	22.1 ± 1.6	18.9–25.2
Training (hours/week)	5 ± 4	1–16	6 ± 3	2–14
Training (km/week)	45 ± 18	20–80	48 ± 19	15–80

**Table 2:** Echocardiographic and Doppler measurements in 2011 and 2021. Data are expressed in mean ± standard deviation.

**Variable**	**Year 2011**	**Year 2021**	***p*-Value**
Heart rate (beats/min)	63 ± 10	66 ± 15	0.282
***End-diastolic measurements:***			
Interventricular septum (mm)	9.6 ± 1.4	10.2 ± 1.8	0.670
Posterior wall (mm)	8.4 ± 1.4	9.3 ± 1.9	0.512
Left ventricular diameter (mm)	51.5 ± 5.4	52.0 ± 6.6	0.435
Aortic diameter (mm)	31.8 ± 4.4	33.4 ± 5.6	0.660
***End-systolic measurements:***			
Left ventricular diameter (mm)	35.0 ± 5.4	34.5 ± 6.2	0.368
Left atrial diameter (mm)	35.6 ± 4.5	37.0 ± 9.6	0.386
Left ventricular ejection fraction (%)	65.3 ± 10.6	67.5 ± 6.7	0.777
***Doppler measurements:***			
Aortic ejection flow (m/s)	1.3 ± 0.4	1.1 ± 0.2	0.093
Aortic velocity–time integral (cm)	20.8 ± 3.1	21.6 ± 4.5	0.302
Mitral E wave (m/s)	0.84 ± 0.15	0.89 ± 0.25	0.337
Mitral A wave (m/s)	0.56 ± 0.15	0.67 ± 0.18	0.666
Mitral E/A ratio	1.59 ± 0.55	1.40 ± 0.50	0.952
Mitral E wave deceleration time (ms)	188 ± 33	203 ± 47	0.076
Average mitral TDI e’ wave (m/s)	0.16 ± 0.03	0.13 ± 0.04	0.454
Average mitral TDI a’ wave (m/s)	0.10 ± 0.03	0.09 ± 0.03	0.823
Average mitral TDI S wave (m/s)	0.11 ± 0.26	0.11 ± 0.29	0.896
Mitral E/e’ ratio	5.6 ± 1.6	6.5 ± 3.5	0.224
Tricuspid TDI e’ wave (m/s)	0.12 ± 0.02	0.12 ± 0.03	0.549
Tricuspid TDI a’ wave (m/s)	0.11 ± 0.03	0.11 ± 0.03	0.110
Tricuspid TDI S wave (m/s)	0.09 ± 0.01	0.09 ± 0.03	0.163
***Two-dimensional strain measurements:***			
Global peak systolic strain (%)	−22.0 ± 2.9	−21.0 ± 2.9	0.192
A3C peak systolic strain (%)	−21.8 ± 3.3	−20.5 ± 3.9	0.979
A4C peak systolic strain (%)	−23.3 ± 2.5	−22.3 ± 2.8	0.075
A2C peak systolic strain (%)	−20.9 ± 2.8	−20.3 ± 2.0	0.307

## Data Availability

Data are available on reasonable request.
